# Presentations of active substance use in the emergency department: A single center’s perspective study in Riyadh, Saudi Arabia

**DOI:** 10.15537/smj.2023.44.2.20220753

**Published:** 2023-02

**Authors:** Mohammed K. Alageel, Alshamoos A. Alwassel, Hamad A. Almohsen

**Affiliations:** *From the Department of Emergency Medicinem (Alageel, Alwassel, Almohsen), College of Medicine, King Saud University Medical City, Riyadh, Kingdom of Saudi Arabia; and from the Department of Emergency Medicine (Alageel), University of British Columbia,Vancouver, Canada.*

**Keywords:** illicit substances, drugs of abuse, clinical presentation, emergency department, Saudi Arabia

## Abstract

**Objectives:**

To explore the most common clinical presentations of active substance users in our institution’s Emergency Department (ED).

**Methods:**

This was a retrospective chart review of all patients that were brought to the ED of King Saud University Medical City in Riyadh, Saudi Arabia thought to be actively using illicit substances, between January 2019 and December 2021. Those with incomplete data were excluded.

**Results:**

A total of 582 patients were included in the study, 532 (91.4%) males, the majority were in the age group 21-30 years old (53.1%). Most patients were fully alert (n=405, 69.6%). Overall, cannabis was used by 349 (60%) of patients. Seventy-four patients presented to the ED because of motor vehicle collisions, the majority were males (98.6%), 35 (47.3%) were the driver of the vehicle and 40 (54.1%) were on cannabis. Males had 5.5 times more medical illness presentations and 10.8 times traumatic illness presentations when compared to females predominantly presenting with psychological illness presentations.

**Conclusion:**

Among Saudi users of illicit substances, the majority were young men with medical illness presentations. The rate of traumatic injuries / vehicular and road traffic accidents is at 15.3%, and cannabis and amphetamine were the most used substances. Screening for active substance use should be conducted using both patient histories and laboratory testing for all high-risk presentations and not solely based on clinical assessment.

**I**llicit substance use and addiction are major causes of medical and social issues and impose a significant financial burden worldwide, as affected patients are among the heaviest users of healthcare resources.^1^ In the course of our everyday work, we frequently encounter active substance users; some of them exhibit overt signs of intoxication, while others are only revealed through interviewing. The literature demonstrates that many emergency departments (ED) patients arrive with undiagnosed active substance use along with a variety of physical and mental disorders, and we see many such persons in our daily practice. These patients are at increased risk for medical issues, trauma, and mental health disease, including disinhibition, a lack of coordination, and poor judgment.^2^

Throughout human history, people have used various chemicals to make them feel happy and euphoric.^3^ However, there are other concomitant effects of this chemical usage, including well-documented harms. Substance use is a global medical, social, and financial issue. In 2017, an estimated 271 million people, aged 15–64 years old, had used illicit substances in the previous year, while 35 million people are estimated to be affected by drug use disorder.^4^ Worldwide, in 2017, there were 585,000 deaths and an estimated 42 million years of “healthy” life lost as a consequence of alcohol and illicit substance use.^5^ Alcohol use is related to 5.3% of worldwide deaths and is implicated in more than 200 medical, mental, and behavioral illnesses as well as injuries.^6^

Substance use in Saudi Arabia raises some unique considerations. It is a predominantly Muslim country, with conservative community values, and any substance that may induce an altered state of consciousness is prohibited.^7,8^ As such, the use of euphoric substances in Muslim communities is considered stigmatizing.^8^ The social implications of drug use are further exacerbated by strict Saudi laws that prosecute substance use and the lack of supportive resources for users and their families.^9^ The associated stigma and fear of prosecution also pose challenges for transparent communication in a clinical setting. With the increasing trend of substance use and the previously noted challenges, it is likely that substance use is mostly underreported in Saudi Arabia.

Studies on substance use in Saudi Arabia are sparse and limited to inpatient settings in substance use treatment facilities. The most commonly used substance is tobacco, followed by amphetamines and khat, while alcohol and heroin use had been decreasing.^9-11^ Patients present to the emergency department (ED) for multiple emergent and non-emergent reasons, with substance use-related visits among the most common. Identifying patients who present to EDs due to substance use directly (such as overdose) or indirectly due to traumatic events (such as motor vehicle accidents involving intoxication) has important implications for identifying patterns of substance use, targeted patient screening, and public health measures. However, the ability of ED practitioners to identify intoxicated patients through clinical assessment is questionable. Observational data suggested that up to 21% of sub-critically injured patients were alcohol-positive, but ED nurses and physicians were only able to identify half of such patients.^12^ Other studies have suggested that specific forms of trauma, such as ocular trauma, were associated with a high incidence of occult substance use, including alcohol and illicit drugs.^13^ Further, a study involving four community hospital EDs found that 11% of injured patients and 5% of deceased patients were positive for alcohol. Alcohol involvement in adolescent trauma has been reported to range from 36% to 48%.^14,15^

The aim of this study is to determine the most common ED clinical presentations of active substance users in Riyadh, Saudi Arabia, compare the results to existing published reports, and explore the types and frequencies of substance use by ED department patients. These observations will shed light on patterns of substance abuse in this high-risk population and, more importantly, identify presentations that are often related to active substance use, helping to inform future harm mitigation interventions.

## Methods

A literature search for related studies was done using PubMed and Google Scholar. This retrospective study was carried out at the ED of King Saud University Medical City (KSUMC), Riyadh, Saudi Arabia. Patients’ files were searched on the local electronic medical records (eSiHi) platform for orders of blood alcohol levels or urine toxicology screening (Dimension® EXL™ THC/amphetamine screen flex). All adult ED patients that had a blood ethanol level or urine toxicology test ordered were screened between January 2019 and December 2021. Patients were considered positive of active use if their laboratory testing was positive in either test or if the clinical documentation was suggestive of active use. We excluded those who had missing presentation data.

Patients’ demographic profile (age and gender), arrival mode to the ED (via ambulance or by car), reason for presentation (medical emergencies, including neurological symptoms, endocrine symptoms, gastrointestinal symptoms, and cardiac symptoms; psychiatric emergencies, including acute psychosis, suicidal attempts, and mood disorders; traumatic emergencies, including falls, motor vehicle collusions, stab wounds, and gunshots), level of consciousness (fully alert, altered or decreased consciousness), time of presentation, day of the week, type of drug used, comorbidities, and disposition were collected.

The research was carried out in accordance with the Helsinki Declaration and Good Clinical Practice Guidelines. The privacy and anonymity of the patients as well as an adequate level confidentiality of the research data were ensured. The study was approved by the Institutional Review Board of the College of Medicine, King Saud University, Riyadh Saudi Arabia (IRB approval no. E-21-5836 IRB KSU).

### Statistical analysis

The statistics we carried out using the Statistical Package for Social Sciences (SPSS) version 25.0 for Windows 11.0 (IBM Corp., Armonk, New York, USA). Results were expressed as numbers and percentages for categorical variables. Univariate and multivariate logistic regression analyses were conducted to determine the significant factors related to the use of illicit substances and their presentation to the ED.

## Results

A total of 582 patients were included in the study: 532 (91.4%) were males, 50 (8.6%) were females, and the majority was in the age group 21-30 years (n=309, 53.1%). Four hundred and sixty-three (79.6%) patients were brought to the ER by private cars, and the majority of them (n=405, 69.6%) were fully alert Glasgow Coma Score (GCS)=15. Two hundred and thirty-eight (40.9%) patients arrived between 23:01 and 7:00 hours, and 408 (70.1%) presented on weekdays (Table 1).

**Table 1:** - Characteristics of the 582 patients brought to the Emergency Department (ED).

Variable	n	%
***Gender***		
Female	50	8.6
Male	532	91.4
***Age in Years***		
<15	5	0.9
15-20	49	8.4
21-30	309	53.1
31-40	139	23.9
41-50	48	8.3
51-60	28	4.8
61-70	3	0.5
71-80	1	0.2
***Mode of Arrival***		
Ambulance	111	19.0
Police vehicle	8	1.4
Private car	463	79.6
***Level of consciousness upon arrival***		
Glass Glow Score <8	34	5.8%
Glass Glow Score (8-14)	143	24.6
Glass Glow Score=15	405	69.6
***Time of arrival/presentation (shift time)***		
7:01 to 15:00 hours	159	27.3
15:01 to 23:00 hours	185	31.8
23:01 to 7:00 hours	238	40.9
***Day of arrival to the ED***		
Weekdays (Sunday to Thursday)	408	70.1
Weekends (Friday and Saturday)	174	29.9

**Figure 1:**
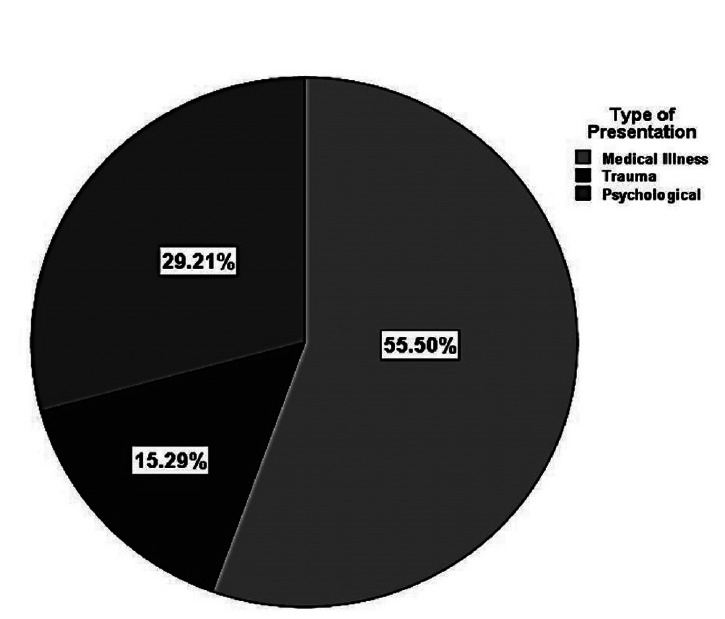
- Distribution of presentations in the sample.

Overall, the most used illicit substance was cannabis (n=351, 60.3%), followed by amphetamines (n=203, 34.9%). One hundred and sixty-two patients (27.8%) used multiple drugs (mean of 1.3 drugs per patient). Figure 1 lists the names and frequencies of the illicit substances used by the 582 patients.

**Figure 2:**
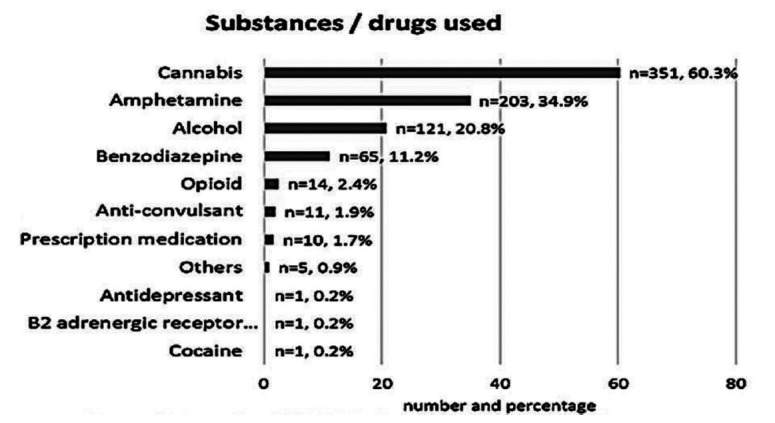
- Names and frequencies of the illicit substances used by the 582 patients.

The most common presentation to the ED was medical illness (Figure 2). Of the 10 substances/drugs identified, amphetamine were used by 62.6%, cannabis by 57.3%, anticonvulsants by 54.5%, and opioids by 50% of patients admitted to the ED for medical reasons. Alcohol (30.6%) and opioids (28,6%) were the most commonly used substances among patients admitted to the ED due to a trauma/vehicular accident, whereas prescription medications were used by 50% of patients admitted/referred to the ED with a psychiatric/psychological presentation, followed by benzodiazepines (35.4%) and cannabis (29.3%).

Of the 89 patients who were brought in due to trauma, 74 were involved in motor vehicular accidents. Of these 74 patients, the large majority were males (n=73, 98.6%) and were between the ages of 21-30 years (n=48, 64.9%). Thirty-five of them (47.3%) were the driver of the vehicle. Forty-seven (63.5%) were brought in on weekdays, while 36 (48.6%) were brought in at night. Sixty-three patients (85.1%) were discharged home, 8 (10.8%) left the ED against medical advice, one (1.4%) was taken into custody by the police, and 2 (2.7%) were transferred to another hospital. More than half of them (n=43, 58.1%) were brought into the ED by an ambulance, and 50 (67.6%) were fully alert. The most commonly used substance among patients who sustained a motor vehicle accident was cannabis (n=40, 54.1%), followed by amphetamines (n=18, 24.3%). Of the 40 motor vehicle collisions (MVC) patients who were using cannabis, 21 (52.5%) were driving the vehicle, while all nine amphetamine users (100%) involved in MVC were driving the vehicle.

Multinomial logistic regression analysis showed that males had more medical illness presentations (adjusted odds ratio [OR]: 5.8, 95% confidence interval [CI]=2.95-11.58, *p*<0.001) and higher traumatic illness risk (adjusted OR: 11.4, 95% CI=2.61-50.04, *p*=0.001) when compared to females. When adjusted to age and gender, the odds that patients will present to the ED with traumatic illness is significantly higher during the hours between 15:00 and 23:00 hours (adjusted OR=2.4, 95% CI=1.22-5.0, *p*=0.011) compared to 7:00 and 15:00 hours and 23:00 and 7:00 hours. A GCS value between 8 and 15 indicated higher risk of medical illness compared to trauma and psychological illness (adjusted OR: 2.2, 95% CI=1.43–3.49, *p*<0.001). Age and presentation day of the week did not show any statistically significant association with the type of presentation (Table 2).

**Table 2:** - Multinomial logistic regression analysis of factors for type of presentation among 582 patients who had substance use.

Type of presentation	Unadjusted				Adjusted		
	Variable	*P*-value	OR	95% CI	*P*-value	OR	95% CI
Medical	***Gender***						
	Male	<0.001	5.5*	2.8 - 10.6	0.001	5.852*	2.95 - 11.58
	Female	-	-	-	-	-	-
Trauma	***Gender***						
	Male	<0.001	10.8*	2.5 - 46.1	0.001	11.488*	2.61 - 50.4
	Female	-	-	-	-	-	-
Medical	***Age***						
	<20	0.553	1.36	0.49 - 3.75	0.559	1.37	0.47 - 3.94
	21-30	0.77	0.886	0.39 - 1.98	0.881	1.067	0.45 - 2.49
	31-40	0.815	1.108	0.47 - 2.61	0.884	1.069	0.43 - 2.62
	41-50	0.769	1.158	0.43 - 3.08	0.745	1.186	0.42 - 3.32
	>50	-	-	-	-	-	-
Trauma	***Age***						
	<20	0.152	3.056	0.66 - 14.07	0.144	3.206	0.67 - 15.32
	21-30	0.347	1.895	0.50 -7.18	0.249	2.23	0.57 - 8.71
	31-40	0.391	1.842	0.45 - 7.44	0.399	1.846	0.44 - 7.67
	41-50	-	-	-	-	-	-
	>50	-	-	-	-	-	-
Medical	***Presentation time***						
	7:00 - 15:00	0.651	0.896	0.55 - 1.43	0.796	0.936	0.56 -1.54
	15:00 - 23:00	0.162	0.734	0.47 - 1.13	0.425	0.829	0.52 - 1.31
	23:00 - 7:00	-	-	-	-	-	-
Trauma	***Presentation time***						
	7:00 - 15:00	0.771	0.914	0.49 - 1.67	0.703	1.133	0.59 - 2.15
	15:00 - 23:00	0.002	2.93*	1.52 - 5.89	0.011	2.44*	1.22 – 5.0
	23:00 - 7:00	-	-	-	-	-	-
Medical	***Day of presentation***						
	Weekdays	0.734	0.931	0.61 - 1.41	0.848	0.958	0.61 - 1.48
	Weekends	-	-	-	-	-	-
Trauma	***Day of presentation***						
	Weekdays	0.12	0.648	0.37 - 1.12	0.151	0.656	0.37 - 1.16
	Weekends	-	-	-	-	-	-
Medical	***GCS***						
	<8	0.151	1.956	0.78 - 4.89	0.126	2.139	0.81 - 5.66
	8-15	0.001	2.105*	1.39 - 3.22	<0.001	2.24*	1.43 - 3.49
	>15	-	-	-	-	-	-
Trauma	***GCS***						
	<8	0.519	0.585	0.11 - 2.98	0.478	0.545	0.10 - 2.92
	8-15	0.435	0.8	0.45 - 1.40	0.263	0.714	0.39 - 1.29
	>15	-	-	-	-	-	-

## Discussion

In this study, we explored how patients who have used narcotics or illicit substances present to the ED. Increased rates of ED visits and admissions related to substance addiction have been documented in previous studies. Since many patients enter the ED with undetected drug and alcohol use, we considered characterizing the different presentations of these patients in an attempt to identify groups with a higher risk of active substance use in the EDs and formulate screening criteria.

According to our findings, young men between the ages of 21 and 30 accounted for most of the patients with a history of substance use or abuse who were admitted to the ED. Of these patients, 60.3% used cannabis, while 34.9% used amphetamines. This supports the conclusion by Bamofleh et al^16^ from 2017 that the most addicted age group in Western Saudi Arabia was 15–25 years old. Alcohol was used less frequently than amphetamines and cannabis. In contrast, ethanol (alcohol) was the most commonly used substance in a survey carried out in the Eastern region of Saudi Arabia in 2020, followed by cannabis (38.6%) and amphetamines (17%).^17^ Further, smaller than the reported rate of 31.5%, 27.8% of our patients showed a bi- or triple-drug use pattern, despite the strict regulations on alcohol and illegal drug usage in Saudi Arabia.^10^ However, only a small number of studies have been published that examined the patterns of drug and illegal substance use in Saudi Arabia and were able to identify the most frequently used types, quantities, administration techniques, and motivations.^9^ According to reports, older Saudi men are more likely to use cannabis, whereas younger men are more likely to use amphetamines.^18^

We found that 74 (12.7%) patients were involved in car accidents, most of whom were men between the ages of 21 and 30 (64.9%). The high prevalence of drug use, combined with risky driving behavior, quantity consumed, and overall drug use, is a strong independent predictor of the number of accidents and injuries.^19^ Our findings are comparable to those of Manimaran et al^20^ who reported that the age range of 21-30 years accounted for 45.7% of MVCs, including alcohol and other drugs. Unsurprisingly, this age group includes people who are just beginning to appreciate their freedom as young adults. Meanwhile, older people who use drugs or abuse substances are more likely to be violent, unemployed, and to physically abuse their spouses.^21^

The finding that men in the ED are 5.5 times more likely than women to present with a medical conditions and suffer serious injuries due to active substance use is noteworthy. In women and girls, the spectrum of substance use is distinct.^22^ Women may report less substance and drug misuse than men, but girls and women report greater rates of all mental health issues and a heavier burden of psychosocial risk factors than men. They are also more likely to need extensive therapeutic treatments. Compared to males, women report more impediments related to family obligations, interpersonal issues, and mental health as well as higher degrees of perceived stigma.^22^

The ongoing issue of substance abuse is still a problem. According to reports, 62% of patients who use or abuse illicit drugs do not have access to services, especially women, who are stigmatized for abusing drugs.^23^ Acute and persistent pain that cannot be controlled, stigmatization and prejudice by medical employees regarding their condition, and hospital restrictions, such as being forbidden to leave the hospital floor, are all reasons why these patients leave the hospital too soon. Moreover, individuals with criminal backgrounds may associate hospitalization with being locked up.^24^

Based on our findings and the risks of medical, traumatic, and psychological sequelae that are likely to occur to patients who abuse drugs and illicit substances, in addition to the need for appropriate interventions and stricter application of the Saudi national program for drug prevention together with widespread community action, as proposed by Mahmoud et al^25^ in 2020, there is also a need for EDs and doctors to provide an environment that is conducive to the safe screening of users of drugs and illicit substances. To ensure effective management, patients who are taking these drugs must be recognized and given priority upon being admitted. Furthermore, patient education should be prioritized for individuals who have a history of substance abuse, overdose, and addiction.^26,27^

### Study limitations

One is the retrospective nature where we have collected data from patients’ files which are subjected to numerous biases thus we were not able to fully elucidate the other potential outcomes including the sequelae, interventions and follow-up management of these patients. Another limitation was the screening assays used by the center in which we conducted this study, as our urine toxicology screening can only detect specific substances (amphetamines, benzodiazepines, opioids, and cannabis). Urine toxicology screening assays have also been shown to have variable sensitivity and specificity due to cross-reactivity with other compounds, and it is likely that we missed patients who were not identified based on their history.^28^ THC may be found in the urine for weeks after use. This may partly explain why THC is much more commonly found that other substances. Moreover, we are unable to extrapolate our findings to the larger community, as the data were collected from a single center in a specific region in Saudi Arabia, without information regarding socioeconomic status or a thorough history of individuals’ substance use patterns or frequencies. Most of our patients were males and we cannot totally deduce that the prevalence of substance abuse among females is low. There may be an underreported high prevalence of substance abuse among the females that needs to be unearthed in future larger studies. Further, there are potential inaccuracies in a retrospective data set from a single institution from a specific region in Saudi Arabia. Therefore, a larger multicenter study with broader screening criteria is needed to identify the prevalence and patterns of active substance use in patients presenting to the ED.

In conclusion, most of the patients who were suspected substance abusers were young to middle-aged men, and majority had a medical issue. As with other studies, cannabis and amphetamine remains to be the most commonly abused substances. As with earlier investigations, most patients in our study showed no overt changes in cognitive status, suggesting that mental status is a poor diagnostic tool for identifying these patients. Even though many individuals showed no outward signs of cognitive impairment, it is important to screen for active drug use. Our findings imply that screening for active substance use should be carried out using both patient histories and laboratory testing for most patients presenting to the ED due to physicians’ limited ability to recognize patients with active substance use based solely on assessment as well as the variety of presentations and age groups presenting with active substance use. To address this issue, national policies and proactive programs are needed in collaboration with public and private organizations
